# Safety and potential benefits of physical therapy in adult patients on extracorporeal membrane oxygenation support: a systematic review

**DOI:** 10.5935/0103-507X.20190017

**Published:** 2019

**Authors:** Daniele da Cunha Ferreira, Miriam Allein Zago Marcolino, Fabrício Edler Macagnan, Rodrigo Della Méa Plentz, Adriana Kessler

**Affiliations:** 1 Residência Multiprofissional Integrada em Saúde com ênfase em Atenção em Terapia Intensiva, Universidade Federal de Ciências da Saúde de Porto Alegre - Porto Alegre (RS), Brasil.; 2 Programa de Pós-Graduação em Ciências da Reabilitação, Universidade Federal de Ciências da Saúde de Porto Alegre - Porto Alegre (RS), Brasil.; 3 Departamento de Fisioterapia, Universidade Federal de Ciências da Saúde de Porto Alegre - Porto Alegre (RS), Brasil.

**Keywords:** Extracorporeal membrane oxygenation, Physical therapy modalities, Physical therapy specialty, Rehabilitation, Early ambulation, Oxigenação por membrana extracorpórea, Modalidades de fisioterapia, Fisioterapia, Reabilitação, Deambulação precoce

## Abstract

Scientific and technological advances, coupled with the work of multidisciplinary teams in intensive care units, have increased the survival of critically ill patients. An essential life support resource used in intensive care is extracorporeal membrane oxygenation. Despite the increased number of studies involving critically ill patients, few studies to date have demonstrated the safety and benefits of physical therapy combined with extracorporeal membrane oxygenation support. This review identified the clinical outcomes of physical therapy in adult patients on extracorporeal membrane oxygenation support by searching the MEDLINE^®^, PEDro, Cochrane CENTRAL, LILACS, and EMBASE databases and by manually searching the references of the articles published until September 2017. The database search retrieved 1,213 studies. Of these studies, 20 were included in this review, with data on 317 subjects (58 in the control group). Twelve studies reported that there were no complications during physical therapy. Cannula fracture during ambulation (one case), thrombus in the return cannula (one case), and leg swelling (one case) were reported in two studies, and desaturation and mild vertigo were reported in two studies. In contrast, improvements in respiratory/pulmonary function, functional capacity, muscle strength (with reduced muscle mass loss), incidence of myopathy, length of hospitalization, and mortality in patients who underwent physical therapy were reported. The analysis of the available data indicates that physical therapy, including early progressive mobilization, standing, ambulation, and breathing techniques, together with extracorporeal membrane oxygenation, is feasible, relatively safe, and potentially beneficial for critically ill adult patients.

## Abstract (pt)

O avanço científico e tecnológico associado à atuação de equipes multidisciplinares nas unidades de terapia intensiva tem aumentado a sobrevida de pacientes críticos. Dentre os recursos de suporte de vida utilizados em terapia intensiva, está a oxigenação por membrana extracorpórea. Apesar das evidências aumentarem, faltam dados para demonstrar a segurança e os benefícios da fisioterapia concomitante ao uso da oxigenação por membrana extracorpórea. Esta revisão reúne as informações disponíveis sobre a repercussão clínica da fisioterapia em adultos submetidos à oxigenação por membrana extracorpórea. A revisão incluiu as bases MEDLINE^®^, PEDro, Cochrane CENTRAL, LILACS e EMBASE, além da busca manual nas referências dos artigos relacionados até setembro de 2017. A busca resultou em 1.213 registros. Vinte estudos foram incluídos, fornecendo dados de 317 indivíduos (58 no grupo controle). Doze estudos não relataram complicações durante a fisioterapia. Fratura da cânula durante a deambulação, trombo na cânula de retorno e hematoma na perna em um paciente cada foram relatados por dois estudos, dessaturação e vertigens leves foram relatadas em dois estudos. Por outro lado, foram feitos relatos de melhora na condição respiratória/pulmonar, capacidade funcional e força muscular, com redução de perda de massa muscular, incidência de miopatia, tempo de internação e mortalidade dos pacientes que realizaram a fisioterapia. Analisando o conjunto das informações disponíveis, pode-se observar que a fisioterapia, incluindo a mobilização precoce progressiva, ortostase, deambulação e técnicas respiratórias, executada de forma simultânea à oxigenação por membrana extracorpórea, é viável, relativamente segura e potencialmente benéfica para adultos em condição clínica extremamente crítica.

## INTRODUCTION

Scientific and technological advances, combined with the work of multidisciplinary teams in intensive care units (ICUs), have increased the survival of critically ill patients. In addition, there has been an increase in the incidence of physical complications due to the deleterious effects of prolonged immobility and the length of invasive mechanical ventilation (MV), contributing to an increase in healthcare costs and mortality, impairment of the quality of life, and lower survival after hospital discharge.^(1)^

One of the advanced features used in ICUs is extracorporeal membrane oxygenation (ECMO), characterized by temporary mechanical support for the heart and lungs^(2)^ in patients with severe respiratory and/or cardiovascular failure refractory to traditional treatment approaches.^(3)^ ECMO can be performed using three types of cannulation: veno-arterial (VA), veno-venous (VV), or venous-arterial-venous (VAV). Regardless of the modality used, large-bore catheters placed in large vessels are connected to a circuit in which blood is pumped into an artificial lung or membrane oxygenator, where oxygen and carbon dioxide are exchanged. In this system, blood is warmed to body temperature before being reinfused into the patient.^(4-7)^

The severe immobility of hospitalized patients with extended ICU stays induces a high degree of muscle mass loss, ranging from 3% to 11% in the first 3 weeks of immobilization.^(8)^ In addition, patients on ECMO support present lower functional capacity, psychological stress, and lower quality of life.^(9)^ The awakening and extubation of these patients are becoming more common, allowing for feeding, communication, active participation in treatment, and incorporation of rehabilitation programs into the hospital routine, helping these patients to maintain muscle strength and function.^(10,11)^

Several protocols of progressive mobilization have been recommended both to rehabilitate^(12)^ and to maintain muscle strength and mass.^(13)^ In this context, physical therapy (PT) is used to reduce the deleterious effects of immobility, stimulate peripheral blood flow, produce anti-inflammatory cytokines, and increase insulin activity and glucose uptake in muscle tissues.^(13)^ However, although the number of studies involving critically ill patients has been increasing, few studies to date have analyzed the safety and potential benefits of PT in adult patients on ECMO support, given the risk of cannula displacement or fracture during cannulation procedures, potentially leading to adverse events. To date, one systematic review was conducted to determine the potential advantages and safety of multimodal PT protocols to improve motor and respiratory function, combined with VV ECMO. The study searched seven databases and included 9 articles published from 2010 to 2014, with a total of 54 participants, including children and adults.^(14)^ The main limitation was the risk of bias related to the types of studies included in the review. Nonetheless, no formal procedure for assessing the methodological quality of these studies was adopted.

In this context, the primary objective of the present systematic review is to determine the safety of PT in adult patients on ECMO support regardless of the type of cannulation used. The secondary objective was to evaluate the potential benefits of this intervention.

## METHODS

This review complied with the recommendations of the Cochrane Collaboration^(15)^ and the Preferred Reporting Items for Systematic Reviews and Meta-Analyses: The PRISMA Statement,^(16)^ and it was recorded in the PROSPERO - International Prospective Register of Systematic Reviews (http://www.crd.york.ac.uk/PROSPERO/) Under No. CRD42017080407.

### Eligibility criteria

Observational studies (cohort, cross-sectional, case control, case report, or case series) that recruited patients aged ≥ 18 years old who were hospitalized in ICUs on ECMO support (regardless of the type of cannulation: VA, VV, or VAV) and who underwent PT using multimodal protocols (respiratory, motor, and/or electrophysical interventions, including light, sound, thermal, or electrical stimulation) during ECMO support were eligible for inclusion. Studies with or without a comparison group were also eligible. However, the comparison groups, when present, had to have undergone ECMO support but not PT. In the case series, the reports of patients younger than 18 years old were excluded. Publications in English, Portuguese, and Spanish were searched.

The safety of PT was the primary outcome of this review and was evaluated according to the mortality rate, adverse events, oxygen perfusion characteristics, hemodynamic stability (oxygen saturation, heart rate, and blood pressure), and other parameters used to describe the clinical status of patients. Secondary outcomes included the length of MV, length of ECMO support, length of ICU stay, and length of hospital stay. Other effects of PT were also identified and described in this review.

### Search strategy

Studies indexed until September 9, 2017, were searched in the MEDLINE^®^ (accessed via PubMed), EMBASE, Cochrane Controlled Trials Register (Cochrane CENTRAL), Latin American and Caribbean Literature in Health Sciences (LILACS), and the Physiotherapy Evidence Database (PEDro) electronic databases. In addition, a manual search was performed on the references of the included studies and published reviews on the subject. The search terms, including indexed terms (MeSH and EMTREE), subject indices, and synonyms, either individually or in combination using Boolean operators (AND and OR), were *‘*Extracorporeal Membrane Oxygenation’, ‘Physical Therapy Modalities’, ‘Rehabilitation’, and ‘Early Ambulation’. Terms related to the outcomes of interest or the type of study were not included to increase the search sensitivity. The date of publication and language restrictions were not included in the search. The complete search strategy used in PubMed is shown in table 1.

**Table 1:** Search strategy using the MEDLINE^®^ database accessed via PubMed

**(#1) Patient**	“Extracorporeal Membrane Oxygenation”[Mesh] OR “Extracorporeal Membrane Oxygenation” [tiab] OR “ECMO Treatment” OR “ECMO Treatments” OR “Treatment, ECMO” OR “Treatments, ECMO” OR “Oxygenation, Extracorporeal Membrane” OR “Extracorporeal Membrane Oxygenations” OR “Membrane Oxygenation, Extracorporeal” OR “Membrane Oxygenations, Extracorporeal” OR “Oxygenations, Extracorporeal Membrane” OR “Extracorporeal Life Support” OR “Extracorporeal Life Supports” OR “Life Support, Extracorporeal” OR “Life Supports, Extracorporeal” OR “Support, Extracorporeal Life” OR “Supports, Extracorporeal Life” OR “ECLS Treatment” OR “ECLS Treatments” OR “Treatment, ECLS” OR “Treatments, ECLS” OR “ECMO” [tiab]
**(#2) Intervention**	“Physical Therapy Modalities”[Mesh] OR “Physical Therapy Modalities”[tiab] OR “Modalities, Physical Therapy” OR “Modality, Physical Therapy” OR “Physical Therapy Modality” OR “Physical Therapy Techniques” OR “Physical Therapy Technique” OR “Techniques, Physical Therapy” OR “Physiotherapy (Techniques)” OR “Physiotherapies (Techniques)” OR “physiotherapy” [tiab] OR “physical therapy” [tiab] OR “Rehabilitation”[Mesh] OR “Rehabilitation” [tiab] OR “Early Ambulation”[Mesh] OR “Early Ambulation”[tiab] or “Accelerated Ambulation” OR “Ambulation, Accelerated” OR “Ambulation, Early” OR “Early Mobilization” OR “Mobilization, Early” OR “respiratory therapy” [tiab] OR “mobilization” [tiab] OR “exercise therapy” [tiab]
**Search**	#1 AND #2

### Study selection and data extraction

After removing duplicates, two independent researchers examined the titles and abstracts of the retrieved articles. Potentially eligible and uncertain studies were selected for independent evaluation of the full text by the same reviewer according to the eligibility criteria. Divergences were resolved by consensus or by a third reviewer. In the case of multiple publications with the same population, the study with the largest sample was selected. Summaries published at conferences were analyzed on a case-by-case basis and were included if sufficient information was available for assessing eligibility. The reviewers were not blinded to the authors or institutions of the studies under review.

After selecting the studies, two independent reviewers collected data using a standard Microsoft Excel spreadsheet (Microsoft Corporation, Redmond, WA, USA). Disagreements were resolved by consensus or by a third reviewer. Data on the number and characteristics of the study populations, ECMO characteristics, comparison groups (when available), intervention protocols, and outcomes were extracted.

### Analysis of the risk of bias

Two investigators independently assessed the risk of bias of the included studies. Descriptive analysis was performed of cohort studies and case-control studies using the Newcastle-Ottawa scale.^(17)^ The Newcastle-Ottawa scale^(17)^ contains eight questions and assesses the methodological quality using a star scoring system based on three criteria: selection, comparability between groups, and the reliability of outcomes or exposures (in cohort or case-control studies, respectively).

Case series and case studies were evaluated with an 18-item scale to assess the quality of the cases series.^(18)^ This 18-item scale was developed using the modified Delphi method, and it was used to assess the clarity with which the data were reported in the studies. The scale was also used to assess study objectives, similarity between cases, outcomes, and conclusions. This tool was developed for case series but was adapted for case studies.

### Data analysis

The included studies did not present sufficient data and were considered very heterogeneous for estimating the occurrence of the outcomes by meta-analysis. Therefore, the extracted data were analyzed qualitatively.

## RESULTS

### Characterization of the studies

Of the 1,208 studies found in the databases and the five studies found in the reference lists, 20 studies^(11,19-37)^ met all of the criteria and were included in this review, providing data on 317 subjects, including 259 patients treated with PT and 58 patients not treated with PT during ECMO support.

**Figure 1:**
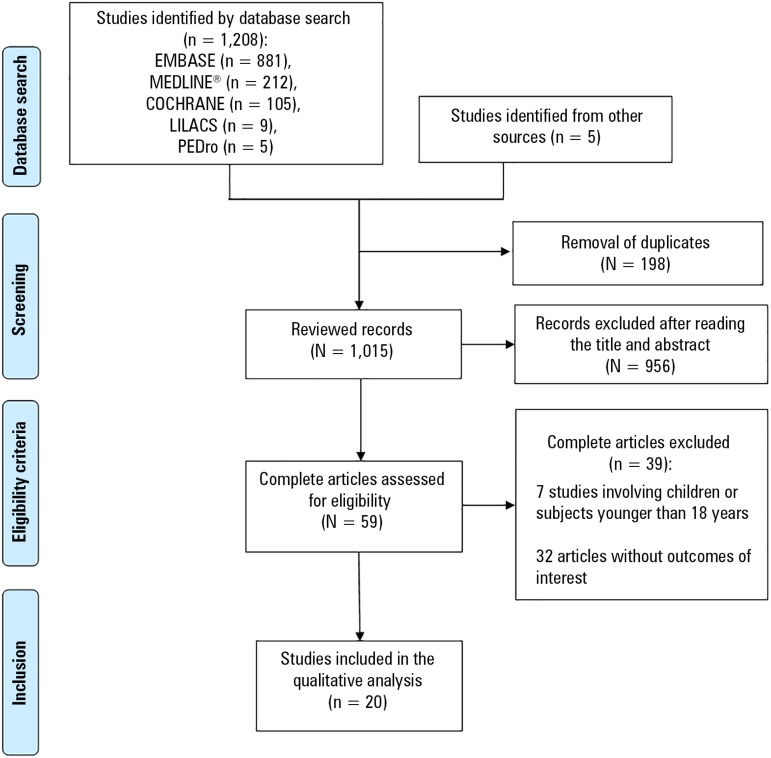
Flowchart of study selection and inclusion. LILACS - Latin American and Caribbean Literature in Health Sciences; PEdro - Physiotherapy Evidence Database.

The study selection process flowchart is shown in figure 1, and a summary of the characteristics of the included studies is shown in table 2.

**Table 2:** Characteristics of the studies included in the review

Study	Type of study	n	Sex	Age (years)*	Reason for ECMO	Type of ECMO	Cannulated vessels	Length of ECMO (days)*	Length of MV (days)	Study category
Abrams et al.^(11)^	Retrospective uncontrolled cohort	IG: 35†	F: 20; M: 15	45.2 ± 18.7	CF (n = 10) ARDS (n = 9) IPD (n = 6) COPD (n = 6) PAH (n = 4)	VV (n = 31) and VA (n = 4)	DL (n = 23); IJV-subclavian artery (n = 4); femoral (n = 8)	NI	NI	Article
Bain et al.^(19)^	Retrospective cohort	IG: 5 CG: 4	F: 6; M: 3	53 ± 22	CF (n = 5); IPF (n = 2); UIP (n = 1); P (n = 1)	VV (n = 9)	NI	IG: 9 (5 - 14) CG: 1.5 (1 - 9)	IG: 12 (5 - 15) CG: 1 (1 - 5)	Article
Carswell et al.^(20)^	Case series	IG: 8	F: 3; M: 5	NI	CF (n = 6); PF (n = 2)	VV (n = 8)	Jugular-femoral; femoral-femoral, or femoral-femoral-jugular	NI	NI	Poster
Cork et al.^(21)^	Case study	IG: 1	M	32	Severe ARF	VV	Jugular-femoral	13	NI	Article
Dennis et al.^(22)^	Retrospective uncontrolled cohort	IG: 18	F: 12; M: 6	49 ± 15	Bridge for LT	VV (n = 18)	Right IJV (DL)	18 ± 16	NI	Poster
Hermens et al.^(23)^	Retrospective uncontrolled cohort	IG: 9	F: 5; M: 4	35 (16 - 59)	CF (n = 7); IPF (n = 1); Lymphangioleiomyomatosis (N = 1)	VV (n = 9)	IJV (DL); femoral-jugular or femoral-femoral	12 (5 - 9)	No patient was on MV	Poster
Keibun^(24)^	Prospective cohort	IG: 10 CG: 13	NI	IG: 60 CG: 61 (mean)	Refractory ARF	VV (n = 31)	NI	NI	NI	Poster
Kikukawa et al.^(25)^	Case study	IG: 1	M	54	ARF due to H1N1	VV	Right IJV-femoral	9	NI	Article
Ko et al.^(26)^	Retrospective uncontrolled cohort	IG: 8	F: 1; M: 7	56.7 ± 10.7	Bridge for LT	VV (n = 7) and VA (n = 1)	Jugular-femoral (n = 7); central (right atrium - ascending aorta) (n = 1)	NI	NI	Article
Kulkarni et al.^(27)^	Case study	IG: 1	M	36	Severe asthma	VV	Right IJV (DL)	5	3	Poster
Morris et al.^(28)^	Case study	IG: 1	F	46	Acute viral interstitial pneumonia	VV	Right IJV-femoral	NI	NI	Poster
Munshi et al.^(29)^	Retrospective cohort	IG: 50/CG: 11	IG: F: 11; M: 39 CG: F: 3; M: 6	IG: 45 ± 14 CG: 44 ± 14	ARDS (80% due to pneumonia)	IG: VV (n = 47) and VA (n = 3) CG: VV (n = 10) and VA (n = 1)	IG: IJV (DL) (n = 26); IJV-femoral (n = 23); three routes (n = 1) CG: IJV (DL) (n = 6); IJV-femoral (n = 4); three routes (n = 1)	IG: 13 (10 - 19) CG: 8 (7 - 10)‡	IG: 3 (0.87 - 7.00) CG: 1.16 (0.33 - 4.00)	Article
Norrenberg et al.^(30)^	Case series	IG: 10	F: 2; M: 8	49 ± 15	NI	VV (n = 5) and VA (n = 5)	Femoral	6 ± 3 days	NI	Poster
Pastva et al.^(31)^	Case study	IG: 1	F	30	CF	VV	IJV (DL)	NI	NI	Poster
Pruijsten et al.^(32)^	Case series	IG: 6	F: 2; M: 4	52 (median)	IPF (n = 2); CF (n = 1); IPD (n = 1); pleuroparenchymal fibroelastosis (n = 1)	VV	Bicaval (DL)	NI	NI	Article
Rahimi et al.^(33)^	Case series	IG: 2 CG: 1	IG: F CG: M	IG: 37 and 25; CG: 23	IG: PF and CF CG: ARDS	VV	IG: Right IJV (DL) CG: Right IJV-femoral	IG: 12 and 4 CG: 30	NI	Article
Rehder et al.^(34)^	Retrospective cohort	IG: 4§ CG: 3§	IG: F: 3; M: 1; CG: F: 1; M: 2	IG: 31 CG: 54.3 (mean)	Bridge for LT	VV	IG: Right IJV (DL) CG: IJV-femoral or femoro-femoral	IG: 8.75 CG: 2.17 (mean)	IG: 1.75 CG: 0.77	Article
Salam et al.^(35)^	Case study	IG: 1	M	55	ARDS	VV	Right IJV (DL)	125	40	Article
Turner et al.^(36)^	Case series	IG: 2§	F	24 and 19	CF and bacteremia + ARF secondary to infection with influenza B	VV	Right IJV (DL)	7 and 14 days	4 and 7	Article
Wells et al.^(37)^	Retrospective cohort	IG: 86 CG: 26	F: 38; M 74	54.9 ± 17.7	Pulmonary embolism, cardiogenic shock; ventricular dysfunction after open heart procedure	VA	Femoral (n = 69) Central (n = 17) NI (n = 26)	NI	NI	Poster

ECMO - extracorporeal membrane oxygenation; IG - intervention group; F - female; M - male; LT - lung transplantation; CF - cystic fibrosis; IPD - idiopathic pulmonary disease; COPD - chronic obstructive pulmonary disease; PAH - pulmonary arterial hypertension; VV - veno-venous; VA - veno-arterial; DL - double lumen cannula; IJV - internal jugular vein; NI - not informed; CG - control group; IPF - idiopathic pulmonary fibrosis; UIP - usual interstitial pneumonia; P - pneumonia; PF - pulmonary fibrosis; ARF - acute respiratory failure; VM - mechanical ventilation; ARDS - acute respiratory distress syndrome.

* Results are expressed as the means ± standard deviations or medians (interquartile ranges) for groups with more than two subjects; the exceptions were identified;

† the study refers to a total cohort of 100 patients, but the results are presented only for the patients who underwent physical therapy;

‡ data reported only on survivors;

§ records of patients younger than 18 years old were excluded.

### Extracorporeal membrane oxygenation support indications, durations, and cannulation strategies

The underlying diseases or clinical conditions that led to the indication of ECMO were cystic fibrosis,^(11,19,20,23,31-33,36)^ pulmonary fibrosis,^(11,19,20,23,31-33,36)^ acute respiratory failure,^(21,24,25,36)^ acute respiratory distress syndrome (ARDS),^(11,29,33,35)^ chronic obstructive pulmonary disease,^(11)^ idiopathic pulmonary disease,^(11)^ pulmonary arterial hypertension,^(11)^ usual interstitial pneumonia,^(19)^ acute viral interstitial pneumonia,^(28)^ pneumonia,^(19)^ bridge to lung transplantation (LT),^(22,26,34)^ asthma,^(27)^ lymphangioleiomyomatosis,^(23)^ pleuroparenchymal fibroelastosis,^(32)^ pulmonary embolism,^(37)^ cardiogenic shock,^(37)^ and ventricular dysfunction after cardiac procedure.^(37)^ One study^(30)^ did not report the reason for using ECMO in the sample (n = 10). The duration of ECMO support was reported in 12 studies^(19,21-23,25,27,29,30,33-36)^ and ranged from 1^(19)^ to 125^(35)^ days.

Fifteen studies^(19-25,27,28,31-36)^ involving 91 patients used VV ECMO, whereas one study^(37)^ involving 112 patients used VA ECMO. Four studies^(11,26,29,30)^ used both VV ECMO (100 patients) and VA ECMO (14 patients), and one of these studies^(29)^ used VAV ECMO in four patients. A total of 191, 126, and four patients underwent VV, VA, and VAV cannulation, respectively. The characteristics of ECMO support are described in table 2.

### Physical therapy techniques

Nineteen studies^(11,19-23,25-37)^ performed physical rehabilitation using different techniques and physical exercises, including active-assisted exercises (17 studies),^(11,19,20,22,23,26-37)^ sitting (12 studies),^(11,20,23,25,26,28,29,32-35,37)^ standing (12 studies),^(11,19,20,22,26,27,29,32,34-37)^ passive mobilization (five studies),^(26,28,29,30,37)^ resistance exercises (four studies),^(23,34-36)^ positioning in bed (one study),^(21)^ stretching (one study),^(34)^ and functional electrical stimulation (FES) of the lower limb muscles combined with cycling (one study).^(31)^ In addition, 93 patients from 11 studies^(11,19,20,22,26,27,32,34-37)^ walked during ECMO support. Keibun^(24)^ reported that the intervention group (IG) (n = 10) underwent PT + VV ECMO. The characteristics of the PT interventions of each study are described in table 3.

**Table 3:** Description of the interventions and outcomes

Study	Intervention	Ambulation	Safety of physical therapy	Number of deaths	Effects of physical therapy
Abrams et al.^(11)^	In-bed active-assisted exercises, in-bed and bedside sitting, and ambulation	Yes	The intervention caused no complications	12	Improvement in functional capacity*
Bain et al.^(19)^	Active rehabilitation and ambulation	Yes	NI	0	The length of MV before LT and ECMO support were significantly greater in the IG than in the CG, whereas the length of MV and the length of ICU stay after LT were significantly shorter in the IG than in the CG*
Carswell et al.^(20)^	Bedside sitting, standing, stationary cycling, gait training, and ambulation	Yes	Desaturation and vertigo during mobilization, recovery with rest after the intervention in some patients	NI	NI
Cork et al.^(21)^	Positioning in bed, hyperinflation with mechanical ventilator, vibration, and aspiration	NI	NI	NI	Favors secretion clearance and pulmonary recovery*
Dennis et al.^(22)^	Bedside standing exercise and ambulation	Yes	The intervention caused no complications	6	Fewer complications associated with immobility*
Hermens et al.^(23)^	Training of lower limb muscles (leg press, in-bed cycling, squatting, and bed-to-chair transfer)	NI	Large swelling (n = 1) and obstructive thrombus in the return cannula (n = 1) after femoro-femoral cannulation	5	Improvement in muscle strength in the lower limbs before LT assessed via the MRC (pre-rehabilitation mean, 3.75; and pretransplantation mean, 4.25)*
Keibun^(24)^	Active rehabilitation	NI	NI	8	Improvement in physical function and decreases in the length of hospital and ICU stay*
Kikukawa et al.^(25)^	Respiratory therapy and bedside sitting	NI	The intervention caused no complications	NI	Improvement in respiratory function*
Ko et al.^(26)^	Passive mobilization, active exercises, FES, bedside sitting, standing, stationary gait training, and ambulation	Yes	Three sessions were interrupted because of tachycardia and tachypnea	NI	Improvement in functionality and fitness*
Kulkarni et al.^(27)^	Active rehabilitation and ambulation (800 feet/day)	Yes	The intervention caused no complications	0	NI
Morris et al.^(28)^	Passive mobilization, bedside sitting, and active exercises	NI	Desaturation during the intervention, which was managed by increasing the blood flow in ECMO. No complications related to cannulation and normal cardiac response to exercise (increase in heart rate and systolic blood pressure) (n = 1)	NI	NI
Munshi et al.^(29)^	Mobilization protocol for patients on ECMO support: passive and active mobilization, bedside sitting, assisted or active standing, stationary gait training, bed-to-chair transfer, corridor ambulation, and treadmill exercise. Patients reached orthostasis	No	The intervention caused no complications	IG: 1 CG 7 (ICU and hospital)	The IG presented lower ICU and hospital mortality and shorter ECMO time*
Norrenberg et al.^(30)^	Mobilization of all joints except for the limb used for ECMO cannulation.	NI	The intervention caused no complications	4	NI
Pastva et al.^(31)^	FES cycling in quadriceps, hamstrings, and buttocks bilaterally, progressive mobilization	NI	The intervention caused no complications	0	Maintenance of the muscle mass † of the rectus femoris (1.5 - 1.6cm) and vastus intermedius (0.95 - 1.15cm) during hospitalization and increase in muscle mass after hospital discharge of more than 2cm in both muscles. Improvement in muscle strength at ICU discharge (MRC sum score of 58/60 and hand grip strength of 60 pounds)*
Pruijsten et al.^(32)^	Bedside sitting, standing, and ambulation	Yes	The intervention caused no complications	2	NI
Rahimi et al.^(33)^	Therapeutic exercises in the supine position and active cycling in bed and assisted bedside sitting.	NI	The intervention caused no complications	1	NI
Rehder et al.^(34)^	Stretching and resisted exercises, sitting, standing, and ambulation (mean distance of 780m)	Yes	The intervention caused no complications	0	Reduction in the MV time after LT and in the total lengths of hospital and ICU stay after LT. None of the IG patients had myopathy after LT, whereas two of the three CG patients presented this complication*
Salam et al.^(35)^	Active exercises with elastic bands, mini-leg press, bedside sitting, and ambulation	Yes	Cannula fracture during ambulation (n = 1)	0	Improvement in fitness before LT*
Turner et al.^(36)^	Resisted exercises, progressive mobilization, gait training, and ambulation	Yes	The intervention caused no complications	0	NI
Wells et al.^(37)^	Functional mobilization, sitting, and ambulation	Yes (n = 5)	The intervention caused no complications	NI	NI

IG - intervention group; MV - mechanical ventilation; ECMO - extracorporeal membrane oxygenation; CG - control group; ICU - intensive care unit; LT - lung transplantation; MRC - Medical Research Council; NI - not informed.

* Potential benefits of physical therapy as reported in the studies;

† Muscle thickness assessed by ultrasonography.

### Safety of physical therapy

#### Adverse events

Among the 20 selected studies, 12 studies^(11,22,25,27,29-34,36,37)^ did not report complications from PT combined with ECMO. Carswell et al.^(20)^ reported that there was a decrease in peripheral oxygen saturation or vertigo during mobilization in some patients, but recovery at rest was rapid. These complications were classified as transient and mild. In the case study of Morris et al.,^(28)^ the decrease in peripheral oxygen saturation was sufficiently compensated for by increased blood flow during ECMO. Ko et al.^(26)^ reported that three therapy sessions were interrupted (without defining the number of patients involved) - one due to tachycardia and two due to tachypnea - during standing or stationary gait training.

One study^(23)^ reported the occurrence of complications from femoro-femoral cannulation (one case that evolved with severe leg swelling and another with an obstructive thrombus in the return cannula). Salam et al.^(35)^ observed the occurrence of cannula fracture during ambulation. Three studies^(19,21,24)^ did not report the safety outcomes of the adopted PT techniques. Therefore, of the 259 patients who underwent PT in the included studies,^(11,19-37)^ four patients^(23,28,35)^ to a maximum of 18 patients (considering all eight patients included in the study by Carswell et al.^(20)^ and that each of the three interrupted sessions in the study by Ko et al.^(26)^ occurred with a different patient) presented adverse events during the interventions. The safety outcomes described in the studies are presented in table 3.

#### Mortality

Eight studies^(11,22-24,29,30,32,33)^ provided data on the number of deaths, which ranged from 1^(33)^ to 16^(29)^ (Table 3). Munshi et al.^(29)^ showed that there was a significant decrease in mortality in patients who underwent PT (IG) compared to those who did not (control group, CG) (odds ratio, 0.19; 95% confidence interval, 0.04 - 0.98), including one death in the IG and seven deaths in the CG. Three other studies^(24,33,34)^ involving patients who did not undergo PT did not present statistical analyses of mortality. The remaining studies^(27,31,34-36)^ reported that the evaluated patients survived after ECMO decannulation.

#### Length of mechanical ventilation

The length of MV before the indication of ECMO was reported in six studies^(19,27,29,34-36)^ and ranged from 0.77 to 151 days (Table 2). Most controlled cohort studies^(19,29,34)^ reported significant differences between the group receiving PT (IG) and the group not receiving PT (CG), and the length of MV in the IG was greater than that in the CG. Rehder et al.^(34)^ reported that the mean MV times in the IG and CG were 1.75 and 0.77 days, respectively. Munshi et al.^(29)^ reported significant differences in the length of MV between the IG and CG (median [interquartile range] of 3 [0.87 - 7.00] and 1.16 [0.33 - 4.00] days, respectively). Bain et al.^(19)^ found that the length of MV was 12 (5 - 15) days in the IG and 1 (1 - 5) day in the CG. One study^(23)^ reported that none of the evaluated patients were on MV when ECMO started, and the remaining 13 studies^(11,20-22,24-26,28,30-33,37)^ did not report the length of MV.

One study indicated that the time of MV after lung transplantation (LT) was shorter in patients who underwent PT + ECMO before LT than in the CG (2 [1 - 5] days and 29 [22 - 54] days, respectively).^(19)^

#### Length of hospital stay

The length of hospital or ICU stay was described in ten studies^(11,19,21,24,25,27,31,34-36)^ (Table 2). Three controlled studies presented the data separated by groups,^(19,24,34)^ and all of the studies reported that the total hospitalization time or length of ICU stay was shorter in the IG. Bain et al.^(19)^ indicated that the length of hospital stay in the IG was shorter than that in the CG (50 [31 - 63] and 94 [51 - 151] days, respectively). In addition, the length of ICU stay after LT was shorter in the IG than in the CG (8 [6 - 22] and 45 [34 - 56] days, respectively).^(19)^

Two studies^(24,34)^ found that PT reduced the length of hospital stay (Table 3). Rehder et al.^(34)^ reported that the mean total hospital stay was 26 days in the IG (n = 4) and 80 days in the CG (n = 3), whereas the mean length of ICU stay was 11 days in the IG and 45 days in the CG. Keibun^(24)^ observed that the mean total hospitalization time was 22 days in the IG (n = 10) and 60 days in the CG (n = 13), and the mean ICU stay was 14 days in the IG and 42 days in the CG. Abrams et al.^(11)^ reported that the lengths of hospital stay in the IG (n = 35) after LT and after ECMO decannulation (mean ± standard deviation) were 34 ± 11 days and 18 ± 17 days, respectively. Nonetheless, these data were not compared with those of the CG. Kikukawa et al.^(25)^ indicated that the evaluated patient stayed 14 days in the ICU and 60 days in the hospital.

### Other effects of physical therapy

In addition to the aforementioned outcomes, ten studies^(11,21-26,31,34,35)^ reported other potentially beneficial effects of PT (Table 3), including secretion clearance; pulmonary recovery;^(21)^ improvement in respiratory function,^(25)^ functional capacity^(11,26,35)^ functionality,^(24)^ and muscle strength;^(23,31)^ maintenance of muscle mass; and decreases in the incidence of myopathy^(34)^ and immobility-associated complications.^(22)^

### Assessment of the methodological quality

Ten cohort studies were scored using the Newcastle-Ottawa scale.^(17)^ The four controlled cohort studies^(19,24,29,38)^ included in this review received three stars. The remaining five studies^(11,22,23,26,37)^ received only two stars. The only parameter not scored in the analyzed studies was the representativeness of the exposed cohort. One study^(29)^ was scored one star for comparability between groups, and it controlled for age when analyzing the cohorts but not for other contributing factors. All of the studies received three stars for the analysis of outcomes, and most of these studies had a retrospective cohort design and reported the occurrence of outcomes in electronic records with no or minimal loss of follow-up of the participants. A detailed evaluation of the risk of bias of the cohort studies is presented in table 1S (Supplementary material).

The methodological quality of the case studies and case series was assessed using an 18-question scale for the case series.^(18)^ Four studies^(32,33,35,36)^ presented good quality in 50% or more of the criteria, whereas one study^(20)^ had low quality in 33% of the criteria. The remaining studies presented low quality in 15% to 25% of the criteria. Ten studies did not present sufficient information to allow for assessing the quality of 20 - 45% of the criteria. One study presented less than 20% uncertainty in the presented information.^(33)^ However, it is worth noting that a scale constructed for case series was used, and after adapting the scale to case studies,^(21,25,27,28,31,35)^ four criteria were not adequate for the type of study and were not scored. The analysis of the risk of bias of the case studies and case series according to each criterion is shown in table S2 (Supplementary material).

## DISCUSSION

The results of the studies listed in this systematic review demonstrate that multimodal PT approaches routinely used in the rehabilitation of adult patients on ECMO support are considered safe because of the absence of severe events and the small number of mild adverse events. Some studies have shown that these interventions might reduce the length of ICU stays and decrease the rate of fatal outcomes, although the probability of reducing mortality has not been confirmed. Furthermore, preventing the deleterious effects of prolonged bed rest has many benefits, including the maintenance and/or gain of muscle strength, together with improved functional capacity relative to individuals who did not undergo PT and decreases in the incidence of myopathy and length of MV after LT. However, the number of these outcomes was not sufficiently large to provide an adequate level of evidence.

With the increasing use of ECMO in patients with potentially reversible acute diseases or as a support strategy (bridge) until the time of lung or cardiopulmonary transplantation, there is a growing need to determine the risk-benefit ratio of PT (early mobilization) in these individuals. The use of ECMO allows for less sedation and anticipates MV weaning in the majority of cases in which clinical stability is reached. Sedatives, even if intermittent, promote delayed ambulation and unnecessary immobilization, leading to physical dysfunction.^(39)^ In turn, early ambulation improves the functional capacity of ICU patients.^(40)^

The literature has indicated that the ambulation of critical patients on MV associated with multimodal PT approaches is safe, improves functional status, and prevents the development of neuromuscular complications.^(41)^ The results of this review demonstrate that PT + ECMO is feasible and safe. Ambulation was possible even in patients with cannulation of lower limb vessels. However, the integration of a multiprofessional team seems to be essential for ensuring the safety and proper monitoring of ventilatory and hemodynamic parameters and for avoiding unnecessary complications and cannula displacement or fracture.^(35)^

Some studies have reported that double lumen cannulas (which reduce problems in the lower limbs) facilitated sitting, standing, bedside exercises, and ambulation,^(11,22,27-29,31-36)^ although femoral cannulation was not considered a contraindication for early mobilization.^(11,20,23,25-30,33,34,37)^

Despite the limited number of controlled studies, the benefits of PT are promising, including decreases in hospital and ICU stays, healthcare costs (22%),^(19)^ length of MV, morbidity and mortality,^(29,34)^ and the incidence of myopathy,^(34)^ as well as an increase in physical capacity.^(24)^

This systematic review is not the first on this subject. However, the study by Polastri et al.^(14)^ had some important limitations. First, the review by Polastri et al.^(14)^ included studies that used only VV ECMO and studies of pediatric patients, for whom rehabilitation programs are distinctive. Second, the search for articles was terminated in 2014, underscoring the need to update the search. Furthermore, it is of note that only observational studies were conducted despite increased interest in the subject. Nevertheless, observational studies cannot fully assess the effects of interventions because of the risk of selection bias and confounding bias, especially in retrospective studies. In contrast, real-life studies provide safety data that can be used as primary information for developing randomized, clinical trials.^(42)^ In nine studies, the only data source included was Congressional abstracts. Despite the limited availability of information in these abstracts, the inclusion of gray literature helps to reduce the effect of publication bias on the results of systematic reviews and reveals underestimated risks in published studies.^(38)^

In addition to observational studies with an inherent risk of bias in the methodology, most of the studies had low methodological quality. Cohort studies^(11,19,22,23,24,26,29,34,37)^ had limitations regarding selection and comparability because the exposed cohort was composed of a specific subgroup of patients, and only four studies had a CG.^(19,24,29,34)^ Nevertheless, the reliability of outcomes was considered high since most of the studies were retrospective, with data extraction from medical records and little loss of follow-up.

The case studies and case series also presented limitations in methodological quality, and only four studies presented good quality in 50% or more of the study criteria.^(32,33,35,36)^ Moreover, the quality of the reports of methods and results was low, and these reports were classified as insufficient or incomplete. It is worth mentioning that, because of the absence of an adequate tool to evaluate case studies, scoring was performed using a scale constructed for case series,^(18)^ which might have underestimated the quality of these studies.

The scarcity of data emphasizes the need for more studies with robust methodological designs and focusing on assessing the risks and benefits of multimodal PT procedures used in the rehabilitation of adults on ECMO support.

## CONCLUSION

This review demonstrated that physical therapy using respiratory techniques, early progressive mobilization (standing and ambulation), and functional electrical stimulation cycling is feasible and safe for patients on extracorporeal membrane oxygenation support regardless of the type of cannulation used. Nonetheless, more clinical studies are needed to confirm the benefits of physical therapy combined with extracorporeal membrane oxygenation regarding the length of hospital stay and mechanical ventilation, mortality, muscle strength, muscle mass, functional capacity, and lung function.
