# Supplementary material for: Safety and potential benefits of physical therapy in adult patients on extracorporeal membrane oxygenation support: a systematic review
Source: Rev Bras Ter Intensiva. 2019 Apr-Jun;31(2):227–39. doi: 10.5935/0103-507X.20190017 (PMC6649220; doi:10.5935/0103-507X.20190017)
Supplement: Supplementary file 1 [file rbti-31-02-0227-suppl1.pdf]

Safety and potential benefits of physical therapy in adult patients on extracorporeal membrane oxygenation support: a systematic review

Segurança e potenciais benefícios da fisioterapia em adultos submetidos ao suporte de vida com oxigenação por membrana extracorpórea: uma revisão sistemática

Daniele da Cunha Ferreira[1], Miriam Allein Zago Marcolino[2], Fabrício Edler Macagnan[2,3], Rodrigo Della Méa Plentz[2,3], Adriana Kessler[1,3]

Table 1 - Assessing the risk of bias of cohort studies using the Newcastle-Ottawa scale

	Selection				Comparability	Outcome		
Study	Representativeness of the exposed cohort	Selection of the unexposed cohort	Analysis of exposure	Outcome of interest absent at baseline	Control for contributing factors	Analysis of outcome	Follow-up sufficiently long for the outcome to occur	Adequacy of cohort follow-up
Abrams et al.[11]			*	*		*	*	*
Bain et al.[19]		*	*	*		*	*	*
Dennis et al.[22]			*	*		*	*	*
Hermens et al.[23]			*	*		*	*	*
Keibun[24]		*	*	*		*	*	*
Ko et al.[26]			*	*		*	*	*
Munshi et al.[29]		*	*	*	*	*	*	*
Rehder et al.[34]		*	*	*		*	*	*
Wells et al.[37]			*	*		*	*	*

* Score of the evaluated criterion.

Table 2S - Assessing the risk of bias of case series and case studies

Study	1	2	3	4	5	6	7	8	9	10	11	12	13	14	15	16	17	18
Carswell et al.[20]	Yes	PD	No	PD	Uncertain	Yes	Yes	No	PD	Uncertain	Uncertain	No	Uncertain	No	No	Yes	PD	No
Cork et al.[21]*	Yes	Yes	No	PD	NA	NA	Yes	No	PD	Uncertain	Uncertain	No	Yes	NA	NA	No	Yes	Yes
Kikukawa et al.[25]*	Yes	Yes	No	PD	NA	NA	Yes	No	PD	Uncertain	Uncertain	No	Yes	NA	NA	Yes	Yes	Yes
Kulkarni et al.[27]*	Yes	Yes	No	PD	NA	NA	Yes	Yes	PD	Uncertain	Uncertain	No	Yes	NA	NA	Yes	Yes	No
Morris et al.[28]*	Uncertain	PD	No	PD	NA	NA	Yes	No	PD	Uncertain	Uncertain	Yes	No	NA	NA	Yes	PD	No
Norrenberg et al.[30]	Yes	PD	No	PD	Yes	Uncertain	Yes	No	Yes	Uncertain	Uncertain	Yes	Uncertain	Yes	Yes	Yes	PD	No
Pastva et al.[31]*	Yes	PD	No	PD	NA	NA	Yes	No	Yes	Uncertain	Uncertain	Yes	No	NA	NA	Yes	PD	No
Pruijsten et al.[32]	Uncertain	Yes	No	Yes	Yes	Yes	Yes	No	PD	Uncertain	Uncertain	No	Yes	Yes	No	Yes	Yes	Yes
Rahimi et al.[33]	Yes	Yes	No	No	Uncertain	Yes	Yes	Yes	Yes	Yes	Uncertain	Uncertain	Yes	Yes	No	Yes	Yes	Yes
Salam et al.[35]*	Yes	Yes	No	PD	NA	NA	Yes	Yes	PD	Uncertain	Uncertain	No	Yes	NA	NA	Yes	Yes	No
Turner et al.[36]	Yes	Yes	No	Yes	Uncertain	Yes	Yes	Yes	Yes	Uncertain	Uncertain	Uncertain	Yes	Yes	No	Yes	Yes	No

1 - Was the objective/hypothesis clearly reported?; 2 - Were the characteristics of the participants described?; 3 - Were the cases collected in more than one center?; 4 - Were the eligibility (for example, inclusion and exclusion) criteria clearly stated?; 5 - Were participants recruited consecutively?; 6 - Did participants enter the study at a similar disease stage?; 7 - Was the intervention of interest clearly described?; 8 - Were additional interventions (cointerventions) reported in the study?; 9 - Were the outcome measures defined a priori?; 10 - Were the relevant outcomes evaluated using appropriate objective and/or subjective criteria?; 11 - Were the statistical tests used to assess the outcomes of interest appropriate?; 12 - Were the relevant outcomes assessed before and after the intervention?; 13 - Was the duration of follow-up reported?; 14 - Were follow-up losses reported?; 15 - Did the study present variability estimates of the analyzed data for the relevant outcomes?; 16 - Were adverse events correlated with the reported intervention?; 17 - Were the conclusions supported by the results?; 18 - Were conflicts of interest and sources of funding reported? PD - partially described; NA - not applicable. * Study with only one case report.
